# Toward a Multifactorial Conception of the Gilles de la Tourette Syndrome and Persistent Chronic Tic Disorder

**DOI:** 10.3390/brainsci7060061

**Published:** 2017-06-02

**Authors:** Marc E. Lavoie, Kieron O’Connor

**Affiliations:** 1 Department of Psychiatry, University of Montréal, Montréal H1N-3V2, Canada; 2 Centre de Recherche de l’Institut Universitaire en Santé Mentale de Montréal, Montréal H1N-3V2, Canada

## Abstract

Despite recent giant leaps in understanding Gilles de la Tourette’s syndrome (now Tourette Disorder in the DSM 5), accurate multi-modal description, rigorous assessment procedures, and the improvement of evidence-based treatment currently pose a considerable challenge. In this context, the current special edition aims to elaborate three important dimensions in Tourette Disorder. Firstly, the effective characterization and etiological basis of the disorder are reviewed, since such characterization impacts accurate assessment. Secondly, subsequent articles cover the comprehensive evaluation and assessment of tic disorders, essential for treatment planning. Thirdly, the final group of articles propose novel and innovative treatment strategies for pharmacologically and behaviorally reducing tic frequency. In the current editorial address, two main issues seem crucial to the development of interventions for Tourette disorder. Primarily, integrating new technology in treatments, while supporting cognitive and behavioral recovery through learning self-controlled strategies. Additionally, the dissemination of study results to frontline resources, needs streamlining and empirically validated treatments for tic disorders should be the subject of knowledge translation to community organizations and be more widely available to the public.

## 1. Tourette Disorder: An Historical Perspective

Jean-Marc Gaspard Itard described tics in a systematic way for the first time in 1825. The latter reports the case of a 26-year-old French noblewoman, the Marquise de Dampierre, who presented involuntary convulsive spasms and contortions at the level of the shoulders, neck, and face. Shortly afterwards, he also reported the presence of “spasms affecting the organs of voice and speech,” and notes the presence of strange screams and senseless words in the absence of a circumscribed mental disorder [1]. He was also the first to hypothesize a possible genetic transmission of that syndrome. Nonetheless, Itard will not pass his family name to this syndrome. The Gilles de la Tourette syndrome (GTS) was named after a French neurologist, who in 1885 described once more the condition of the Marquise de Dampierre, now aged 86 years old, who continued to make abrupt movements and sounds also known as tics. The same year, Tourette described eight other patients with motor and vocal tics, some of whom had echo phenomena (a tendency to repeat things said to them) and coprolalia (utterances of obscene phrases) [2], which were consistent with similar clinical observations from American neurologists one year later [3]. In a doctoral dissertation published under the supervision of Tourette and Charcot, Jacques Catrou documented 26 similar cases [4] with more details. The merit of Gilles de la Tourette’s report consisted not only in gathering remarkable clinical descriptions of the symptoms that were little documented until then, but also in describing the fluctuating evolution of that syndrome.

Subsequently, there were only few systematic investigations, clinical observations, or particular etiological developments during the first half of the twentieth century. Rather, during this period, a psychoanalytic explanation prevailed, with little or no notable empirical support [5,6,7,8]. Next, in the 1960s, an experimental drug treatment (i.e., haloperidol) surfaced for tics [9]. These results encouraged clinical trials in the United States, which further supported the beneficial effects of neuroleptics [10,11,12]. These pivotal investigations instigated the pursuit to find a useful pharmacological treatment, and therefore the search for a neurobiological etiology, relegating to the background both the psychoanalytic and the behavioral approaches [12,13]. At the present time, pharmacological agents remain the first line of treatment, and still represent the intervention of choice to help people with moderate-to-severe symptoms. Neuroleptic and antihypertensive drugs are currently the favored medications for the management of tics [14,15,16,17]. Other cognitive-behavioral with integrated psychophysiological approaches are also considered as efficient for the treatment of tics [18]. New neuro-stimulation approaches are also emerging, with transcranial magnetic stimulation, direct current stimulation, or deep-brain stimulation.

## 2. Etiology and Characterization of Tic Disorders

From the beginning, the description by Tourette referred to the nervous origin of the tics without presenting an explanatory link. With the advent of neuroimaging and modern psychopharmacology, new hypotheses emerged at the end of the 20th century. Current studies using magnetic resonance imaging (MRI) and positron emission tomography (PET) have consistently reported volumetric and metabolic reductions in lentiform [19,20,21,22] and caudate nuclei in childhood [23] that could predict the severity of tic and obsessive-compulsive symptoms in early adulthood [24]. Of course, the basal ganglia are not the sole cerebral structures involved in the pathogenesis of GTS. An extensive investigation [25] comparing a large sample of GTS and controls aged 6–63 years old showed increased volumes of the head and medial surface of the hippocampus and the dorsal and ventral surfaces of the amygdala. Volumes of these subregions declined with age in the GTS group but not in controls, so the sub-regions were larger in GTS children, but significantly smaller in GTS adults than in the control group. In children and adults, volumes in these subregions correlated inversely with the severity of tic, suggesting that enlargement of these structures has a neuro-modulatory effect on tics. In addition to these networks, motor and sensorimotor cortices have shown metabolic increases associated with heightened activation in premotor cortex and supplementary motor area (SMA) with PET imaging [20,22,26,27]. Cortical thinning in sensorimotor areas was also correlated with tic severity and was most prominent in ventral portions of the homunculi that control the facial, orolingual, and laryngeal muscles commonly involved in tic expressions [28,29].

Motor and sensorimotor cortices have also shown metabolic increases associated with heightened activation in the premotor cortex and supplementary motor area. More precisely, altered microstructural changes in white matter were detected in the precentral gyrus, a brain region involved in sensorimotor processing [30,31,32]. Based on these results, tic symptoms in adults could be mainly caused by alterations in prefrontal areas, thalamus and putamen, while changes in the cingulate gyrus could reflect secondary compensatory mechanisms.

## 3. Clinical Assessment and Epidemiology of Tic Disorders

It is noteworthy that the psychiatric criteria in the last edition of the Diagnostic and Statistical Manual (DSM) for the Gilles de la Tourette syndrome were relatively comparable to the 19th century original description. The syndrome is currently classified in the DSM5 (APA 2013) with motor disorders listed among the neurodevelopmental disorder category. The essential features are the presence of simple or complex multiple motor tics and one or more vocal tics. Simple tics are defined as non-voluntary repetitive contractions of functionally related groups of skeletal muscles in one or more parts of the body including blinking, cheek twitches, and head jerks, among others [33]. Complex tics may take the form of self-inflicted repetitive actions such as nail biting, hair pulling, head slapping, teeth grinding, or tense–release hand gripping cycles. The tics appear many times a day with onset longer than a year and prior to 18 years old. Depending on the sample characteristics, between 0.15% and 1.1% of all children had GTS, and boys outnumbered girls by at least 4:1 [34], with the most severe period of tic severity occurring at 10 years old [35], followed by a decrease until adult age with approximately 40% eventually becoming symptom-free [36].

## 4. Managing Tics and Habits with the Cognitive-Behavioral Approach

Azrin and Nunn [37] were the first researchers to suggest that tics could be prevented once they onset by other behaviors. In their book titled *Habit Control in a Day*, they suggested that the tic could be prevented by activating a response antagonistic to the tic and tensing the opposite muscles, so impeding the tic. With practice, the tic could be controlled until the tic was countermanded and the person practiced this antagonist response when they detected tic onset. This habit reversal technique has evolved and has recently been developed into a comprehensive behavioral intervention for tics (CBIT) where the main component is a competing response awareness and analysis of the stimulus and consequences of ticking in order to manage contingencies to reward non-ticking. There have been several studies affirming the validity of the CBIT approach, and there are published manuals [38]. Another behavioral approach to reducing tics is exposure and response prevention, where the client tolerates the urge to tic whilst resisting the action of ticking [39]. Although this treatment seems especially effective with severe tics not amenable to other approaches, not all people with tic disorder experience premonitory urges [40]. These behavioral approaches address the tic when it has occurred or when it is about to occur. The cognitive psychophysiological (CoPs) approach to management can be used in conjunction with the CBIT, but rather aims at regulating the high level of sensorimotor activation present in these populations and preventing the buildup of tension that leads to tic bursts [18,41,42,43]. Although empirical studies are limited, the CoPs approach has also shown effectiveness in treating adults affected by tic disorder [44]. The CoPs approach draws on cognitive behavioral and psychophysiological manifestations of motor activation in GTS, some of which are reviewed in this volume with the aim of linking the multi-level processes evoking tic onset with behavioral management procedures. These experimental and clinical findings have led to the elaboration of the CoPs treatment model [44] which proposes that: (1) an overactive style of planning in tic disorders prevents optimal preparation for action; (2) this style leads to problems regulating arousal/inhibition processes, particularly under circumstances where regulation is open-looped, controlled, and has unpredictable parameters; (3) such high levels of motor activation create tension and frustration and are likely to evoke ticking, which addresses the cognitive psychophysiological sources of motor activation, reducing background tension and preventing tic onset. In targeting cognitive-behavioral and physiological sources creating tension, there were also significant changes post-treatment in measures of self-esteem, anxiety, depression and style of planning action. More recent clinical trials have adapted CoPs for children [45]. The CoPs therapy continues to evolve, but emphasizes the benefits of integrating multimodal findings into evaluation and treatment.

## 5. New Treatment of Tic Disorders

Various treatments have been proposed to help patients, but the majority of prescription drugs as much among adults as among children showed a variable response, even sometimes on the same individual. Because of the dominant hypothesis of tics as a problem of the motor circuit and the dopaminergic system, dopamine antagonistic neuroleptics are regularly the main treatment. Therefore, many researchers have initially observed that pharmacological agents that trigger an increase in dopaminergic functions exacerbate tics [46,47,48], whereas those that bring a decrease (antagonist) of the dopaminergic action tends to reduce the tic frequency [49,50].

Among children and teenagers, original controlled trials have shown that the frequency of tics decreases by 50% after the use of haloperidol or pimozide [51]. However, typical antipsychotics like Haldol may cause extrapyramidal symptoms, characterized by involuntary movements, impatience, a need to constantly move, and significant trembling, among other symptoms. Atypical drug therapy or drug combinations are retained for more complex cases as well as in the presence of associated disorders. However, side effects also occur in approximately 80% of individuals, and only 20–30% of patients afflicted with GTS continue pharmacological treatment for an extended period [52]. The effectiveness of risperidone (atypical neuroleptic) has progressively been proven to reduce tics, despite the possibility of significant long-term side effects, such as an increased risk of hyperglycemia and diabetes [53]. Other pharmacological agents (antidepressants or other neuroleptics) can provide positive results in reducing tics, but these results are often inconsistent and generally come from unique cases or non-randomized trials [54]. There is also a newer class of drugs that diminish presynaptic dopamine by inhibiting vesicular monoamine transporter type 2 (VMAT2), such as tetrabenazine [55], and the emergence of other VMAT2 inhibitors, including deutetrabenazine and valbenazine [56], that could show a better pharmacologic and side effect profile for the treatment of patients with Tourette disorder. Hence, targeted neurostimulation such as the repetitive transcranial magnetic stimulation and the tDCS, as well as the deep brain stimulation [57] also showed promising results for the treatment of refractory GTS [58].

## 6. Conclusions and Contemporary Questions

GTS is a complex neuropsychiatric disorder that affects more people than previously thought. In the last decade, past research has made progress in the treatment of this syndrome, but many questions remain open. Why have many patients failed to respond to current treatment? Why are they often misdiagnosed? Are the symptoms really disappearing in adults? These questions can only be approached with a multidisciplinary angle combining neurology, developmental psychology, psychopharmacology, genetics and psychiatry. So, a uni-disciplinary approach disallows integration of the cognitive, structural, and functional levels of cerebral functioning. Structured interviews are valuable to follow up on clinical states, but they only yield superficial or indirect information on the brain functioning. A coherent model of GTS from a single approach is unlikely, since this pathology is multifaceted. A cognitive-behavioral approach links impairments with the clinical expression of the illness that will impact on therapeutic strategies. However, it provides little information about the cerebral roots of the disease. Neuropsychology allows valid inferences about discrete anomalies, but inferences are mainly based on our knowledge of focal lesions, not on functional disorders. Brain imaging is appropriate for identifying localized metabolic abnormalities. However, it is limited by its low temporal resolution that does not take account of the real-time dynamics of the neurocognitive mechanisms involved in the cascade of information processing [59]. The event-related potentials (ERP) approach has shown encouraging promise to decipher the furtive stream of information processing and relate it to electrocortical functioning in GTS, but the potential aberrant source generators of that electro-cortical activity remain elusive because of the low spatial resolution of ERP techniques. In addition, other concomitant symptoms are often underestimated in populations with Gilles de la Tourette syndrome, leading to incorrect diagnosis or treatment. The potential benefit of the current approach will be to extract a complete profile allowing prediction of symptom development or treatment success.

From a clinical perspective, effective and individualized therapeutic action should not only include the modification of motor symptoms, but should also include cognitive strategies to deal with tics. It is necessary to broaden our conception of GTS in order to see it not only as a neurological, but also as a psycho-physiological syndrome, because a multifactorial treatment induces a maximal effect on many levels and helps to decrease and to better manage the frequency and intensity of the symptoms. Nonetheless, this approach needs to combine both cognitive and behavioral perspectives, while taking into account physiological aspects that can potentially exacerbate the behavioral reaction.

In conclusion, two considerations seem fundamental to the development of specialized interventions for GTS in the near future. First, integrating new technology as an instrument of treatment: these new possibilities can support cognitive and behavioral recovery through learning self-controlled strategies. Second, the dissemination of study results to frontline resources needs streamlining. Finally, treatments for tic disorders which are empirically validated should be the subject of knowledge translation to community organizations and be more widely available to the public.
